# Baseline Brain Activity Changes in Patients With Single and Relapsing Optic Neuritis

**DOI:** 10.3389/fnhum.2018.00144

**Published:** 2018-04-20

**Authors:** Zhuoqiong Ren, Yaou Liu, Kuncheng Li, Yunyun Duan, Huang Jing, Peipeng Liang, Zheng Sun, Xiaojun Zhang, Bei Mao

**Affiliations:** ^1^Xuanwu Hospital, Capital Medical University, Beijing, China; ^2^Beijing Key Laboratory of Magnetic Resonance Imaging and Brain Informatics, Capital Medical University, Beijing, China; ^3^Beijing Tongren Hospital, Beijing, China

**Keywords:** resting-state, fMRI, ALFF, optic neuritis, spontaneous activity

## Abstract

**Purpose**: To investigate spontaneous brain activity amplitude alterations in single and relapsing optic neuritis (sON and rON, respectively) and their relationships with clinical variables.

**Methods**: In total, 42 patients with sON, 35 patients with rON and 50 healthy volunteers were recruited. Resting-state functional Magnetic Resonance Imaging (rs-fMRI) scans were acquired for all participants and compared to investigate the changes in the amplitude of low-frequency fluctuations (ALFFs) among the three groups. The relationships between the ALFFs in regions with significant differences in the groups and clinical variables, including the logarithm of minimal angle of resolution (LogMAR), Expanded Disability Status Scale (EDSS) score and disease duration, were further explored.

**Results**: Compared with healthy volunteers, the sON and rON patients showed significantly decreased ALFFs in several regions of the occipital and temporal lobes (i.e., inferior occipital gyrus and superior temporal gyrus; corrected *p* < 0.01 using AlphaSim). The sON patients showed significantly increased ALFFs in the left caudate and certain regions in the frontal lobes (i.e., medial frontal gyrus), whereas the rON patients showed increased ALFFs in the bilateral inferior temporal gyrus and left medial frontal gyrus (corrected *p* < 0.01 using AlphaSim). Significantly decreased ALFFs were observed in the right inferior parietal lobule (IPL), left posterior cingulate and precuneus in the rON patients compared with those in the sON patients (corrected *p* < 0.01 using AlphaSim). Significant correlations were observed between the disease duration and ALFF in the left middle temporal gyrus, left inferior occipital gyrus, right lingual gyrus and right IPL (*p* < 0.05).

**Conclusion**: Functional impairment and adaptation occurred in both the sON and rON patients. Impairment mainly involved the occipital cortex, and functional adaptions predominantly occurred in the frontal lobe. Functional damage was more severe in the rON patients than in the sON patients and correlated with the disease duration.

## Introduction

Optic neuritis (ON) is a demyelinating inflammatory disease of the optic nerve, and it is strongly associated with multiple sclerosis (MS; Hickman et al., 2002; Miller et al., 2005) and neuromyelitis optica (NMO, also called Devic’s disease; Wingerchuk et al., 1999). ON results in loss of vision and a conduction block (Youl et al., 1991; Guy et al., 1992; Toosy et al., 2014). As a common type of ON, single optic neuritis (sON) is considered a clinical demyelinating event that is isolated in time and could be the initial presentation of MS or NMO. Relapsing optic neuritis (rON), including relapsing inflammatory optic neuritis (RION) and chronic relapsing inflammatory optic neuritis (CRION; Benoilid et al., 2014), is more variable in both visual recovery and neurological prognosis (Toosy et al., 2014).

To date, the most valuable predictor for the development of subsequent clinically definite MS (CDMS) is the presence of white matter (WM) abnormalities on brain MRIs corresponding to demyelinating lesions (Balcer, 2006; Shams and Plant, 2009; Dooley and Foroozan, 2010; Menon et al., 2011). However, most ON patients, particularly clinically isolated ON patients, do not present evidence of brain lesions in conventional brain MRI scans. Thus, a new method that can be used to evaluate brain changes will have a tremendous impact on patient healthcare. Resting-state functional MRI (rs-fMRI) can reflect the baseline brain activity (Biswal, 2012). Previous fMRI studies have revealed decreased functional connectivity in the visual system after acute ON (Wu et al., 2015) and dysfunction in the default-mode network, cerebellum, and limbic system in ON (Huang et al., 2015). Low-frequency fluctuation (LFF) could reflect low-frequency neural oscillation both for spontaneous and evoked brain activity. For example, steady-state BOLD responses (SSBRs) has been investigated to reflect the evoked fluctuation by cognitive task, which is independent of the neurovascular coupling and represents the underlying process of neural oscillation (Wang et al., 2014, 2015, 2016, 2018). As to the resting state, the amplitude of low-frequency fluctuation (ALFF), an alternative index in which the square root of power spectrum was integrated in a low-frequency range, has been suggested to reflect the intensity of regional spontaneous brain activity (Kiviniemi et al., 2000; Zang et al., 2007), thus, it is considered as a measurement of the amplitude of regional spontaneous brain activity. Recently, it has already been applied in several areas of neuroscience and neurological diseases (Liu et al., 2011a,b, 2012; Kwak et al., 2012; Turner et al., 2013). Abnormal brain ALFF has been found in MS (Liu et al., 2011b) and NMO (Liu et al., 2011a) and even in clinically isolated syndrome (CIS) patients (Liu et al., 2012). However, the mechanisms underlying the baseline brain activity changes in sON and rON have not been clarified. This study aimed to investigate the brain activity changes of sON and rON patients and correlate the functional changes with clinical variables.

## Subjects and Methods

### Subjects

We recruited 77 patients with ON, including 42 patients with sON (7 males, 35 females; mean age 36.0, age range 15–58 years) and 35 patients with rON (11 males, 24 females; mean age 34.4, age range 16–58 years), and 50 age- and sex-matched healthy controls (HCs; 16 males, 34 females; mean age 33.7, age range 19–55 years). The main demographic and clinical characteristics are reported in Table 1. A diagnosis of ON was performed by an experienced neurologist according to the standard clinical criteria (Beck et al., 1992). Inclusion criteria were: (1) acute vision loss with or without eye pain; (2) different types of visual field defects; (3) normal or swollen optic nerve head; (4) no deterioration in vision after steroid discontinuation; (5) no previous history of MS or NMO and no previous neurological symptoms; (6) no other diseases causing vision loss, such as vascular problems, optic space-occupying lesions, etc.; (7) fat-suppressed orbital MRI showing lesions or atrophy of the optic nerve; and (8) no typical demyelinating lesions in the brain. In the sON group, 34 patients had unilateral ON and eight patients had bilateral ON. The standardization logarithm of minimal angle of resolution (LogMAR) visual score of the affected eye was 0.92 ± 0.96 (mean ± SD). In the rON group, 21 patients suffered from unilateral ON and 14 presented bilateral ON. The range of relapsing times were from 2 to 12, with an average of 3.1 (±2.3) relapses. The standardization LogMAR visual score of an affected eye on MRI scan was 0.77 ± 0.90 (mean ± SD). The subjects were all right-handed as measured by the Edinburgh inventory (Oldfield, 1971). The HCs were free of any history of neurological disease and presented a normal ophthalmologic examination, naked eye or corrected visual acuity (VA) of 1.0 and normal findings on conventional MRI examination. The institutional review board of Xuanwu Hospital, Capital Medical University approved the study, and written informed consent was obtained from each participant.

**Table 1:** Descriptive data of the study groups.

	sON	rON	Controls	*P* value
Number of subjects	42	35	50	
Mean age (range) [years]	36.0 (15–58)	34.4 (16–58)	33.7 (19–55)	0.221^a^
Sex (M/F)	7/35	11/24	16/34	0.195^b^
Median disease duration (range) [months]	1.3 (0.2–4)	45.2 (1–240)	–	<0.001^c^
EDSS (Visual function)	3.14 ± 1.32	3.94 ± 1.77	–	0.030^c^
LogMAR scores	0.92 ± 0.96	0.77 ± 0.90	–	0.479^c^

*sON, single optic neuritis; rON, relapsing optic neuritis; EDSS, Expanded Disability Status Scale. ^a^x2 test. ^b^Main effect of group in the analysis of variance. ^c^Two-sample t-test*.

### Data Acquisition

All MR measurements were performed with a 3.0-T MR system (Magnetom Trio Tim; Siemens, Erlangen, Germany) using an 8-channel head coil in the Radiology Department of Xuanwu Hospital, Capital Medical University. During the rs-fMRI, subjects were instructed to keep their eyes closed and not to move or think of anything in particular. A standard head coil was used with foam padding to restrict head motion. All axial slices were positioned parallel to a line that joins the AC-PC line of the corpus callosum. Axial T2-weighted turbo spin-echo and fluid-attenuated inversion recovery sequences were performed for WM lesion detection (35 axial slices, TR/TE = 5000 ms/87 ms; field of view (FOV) = 256 mm × 256 mm; voxel size = 1 mm × 1 mm × 4 mm, slice thickness = 4 mm). Functional data were acquired with a gradient-echo echo-planar sequence (GRE-EPI) sensitive to Blood Oxygen Level Dependent (BOLD) contrast to acquire 180 functional images (TR/TE = 2000 ms/30 ms; flip angle = 90°; field of view (FOV) = 220 mm × 220 mm; voxel size = 3.4 mm × 3.4 mm × 3 mm; slice thickness = 3 mm; gap = 1 mm).

### Data Preprocessing

Image preprocessing was conducted using Statistical Parametric Mapping (SPM5^1^). The data for each fMRI scan contained 180-time points. The first 10 volumes of the functional images were discarded for signal equilibration. The remaining fMRI images were corrected for the acquisition delay between slices and for head motion. All participants had less than 1.5 mm maximum displacement in the x, y, or z plane and 1.5° of angular motion during the whole fMRI scan. Then, images were normalized to the standard SPM5 echo-planar imaging template and resampled to 3 mm cubic voxels. Next, the Resting-State fMRI Data Analysis Toolkit^2^ was used to remove the linear trend of time courses and perform temporal bandpass filtering (0.01–0.08 Hz). The resulting data were spatially smoothed (4 mm FWHM Gaussian kernel).

### ALFF Analysis

An ALFF analysis was performed using the Resting-State fMRI Data Analysis Toolkit^2^. The procedure for calculating the ALFF is similar to that described in previous studies (Liu et al., 2011a,b, 2012). The filtered time series was transformed to a frequency domain with a fast Fourier transform (FFT; parameters: taper percent = 0, FFT length = shortest), and the power spectrum was then obtained. The square root was thus calculated at each frequency of the power spectrum, and the averaged square root was obtained across 0.01–0.08 Hz at each voxel. According to Zang et al. (2007), in the present study, the ALFF of each voxel was divided by the individual global mean of ALFF within a brain-mask which is obtained by removing the tissues outside the brain.

### Statistical Analysis

We performed an analyses of variance (ANOVA) to investigate the ALFF differences between the two groups of ON patients and HCs using the SPM Toolbox, with age and gender included as covariates. The voxels with a combined threshold of *p* < 0.005 and cluster size ≥270 mm^3^ (10 voxels) were considered to show a significant difference between the two groups. A corrected threshold of *p* < 0.01 was determined by Monte Carlo simulations using the AlphaSim program (Parameters were a FWHM = 4 mm and a mask of the whole brain gray matter tissue)^3^. The significantly different brain regions were superimposed on the standard T1-weighted imaging of the Montreal Neurological Institute (MNI). Correlative analyses via MATLAB were performed to explore the relationships between the Expanded Disability Status Scale (EDSS) scores, disease duration, LogMAR and ALFF values presenting significant group differences.

## Results

### Demographic and Clinical Characteristics

As shown in Table 1, no significant differences were observed in age and gender among the three groups (HCs, sON and rON). The rON patients showed higher EDSS than sON patients (*P* = 0.03). The sON and rON patient group had an average disease duration of 1.3 (range from 0.2 to 4) months and 45.2 (range from 1 to 240) months, respectively.

### Differences in ALFFs Between ON Patients and HCs

**Figure 1:**
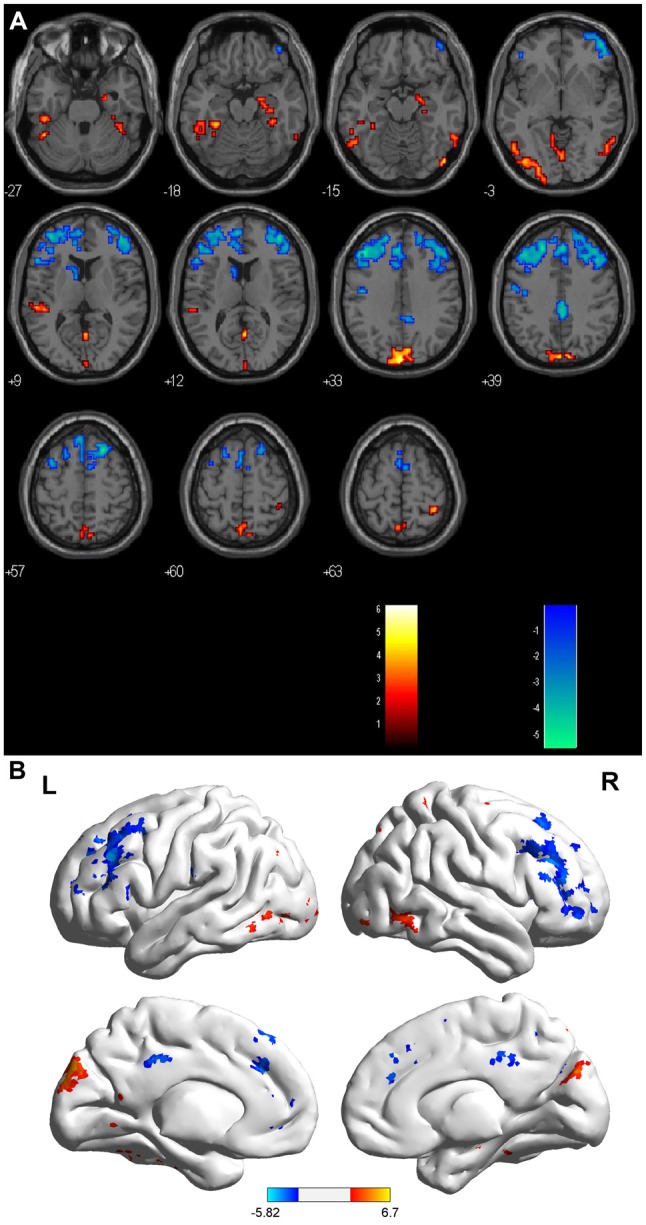
**(A,B)** ALFF: RED = NC > sON; BLUE = sON > NC. Brain areas showing significant ALFF differences between the single optic neuritis (sON) patients and normal controls. Notes: the different brain regions include the bilateral cuneus/precuneus, bilateral fusiform gyrus, bilateral inferior occipital gyrus, right posterior cingulate, right parahippocampal gyrus/hippocampus, right postcentral gyrus and left superior temporal gyrus, left middle frontal gyrus, right superior frontal gyrus, right middle cingulate gyrus, left medial frontal gyrus, left precentral gyrus and left caudate. The red areas represent lower ALFF brain regions, and the blue areas represent higher ALFF brain regions (corrected *p* < 0.01 using AlphaSim). Abbreviations: ON, optic neuritis; ALFF, amplitude of low-frequency fluctuation; L, left; R, right.

**Figure 2:**
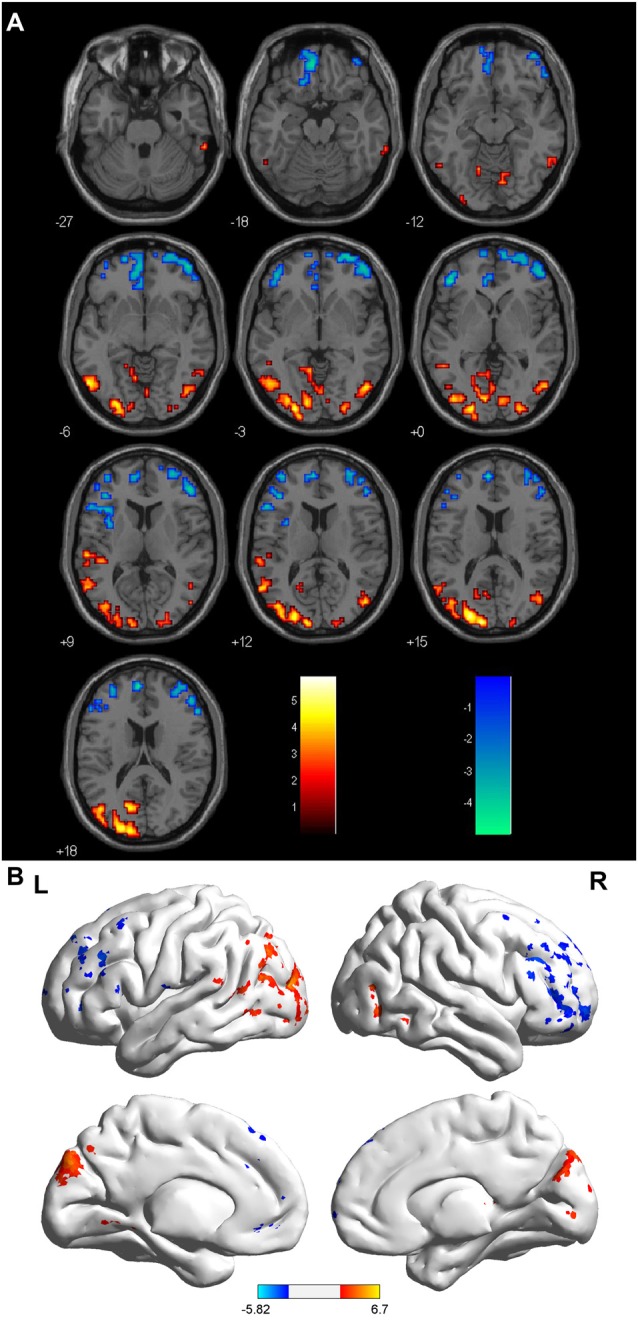
**(A,B)** ALFF: RED = NC > rON; BLUE = rON > NC. Brain areas showing significant ALFF differences between the relapsing optic neuritis (rON) patients and normal controls. Notes: the different brain regions include the left cuneus/precuneus, right inferior temporal gyrus, left inferior temporal gyrus, bilateral lingual gyrus, right middle occipital gyrus, left superior temporal gyrus, and bilateral inferior and medial frontal gyri. The red areas represent lower ALFF brain regions, and the blue areas represent higher ALFF brain regions (corrected *p* < 0.01 using AlphaSim). Abbreviations: ON, optic neuritis; ALFF, amplitude of low-frequency fluctuation; L, left; R, right.

**Figure 3:**
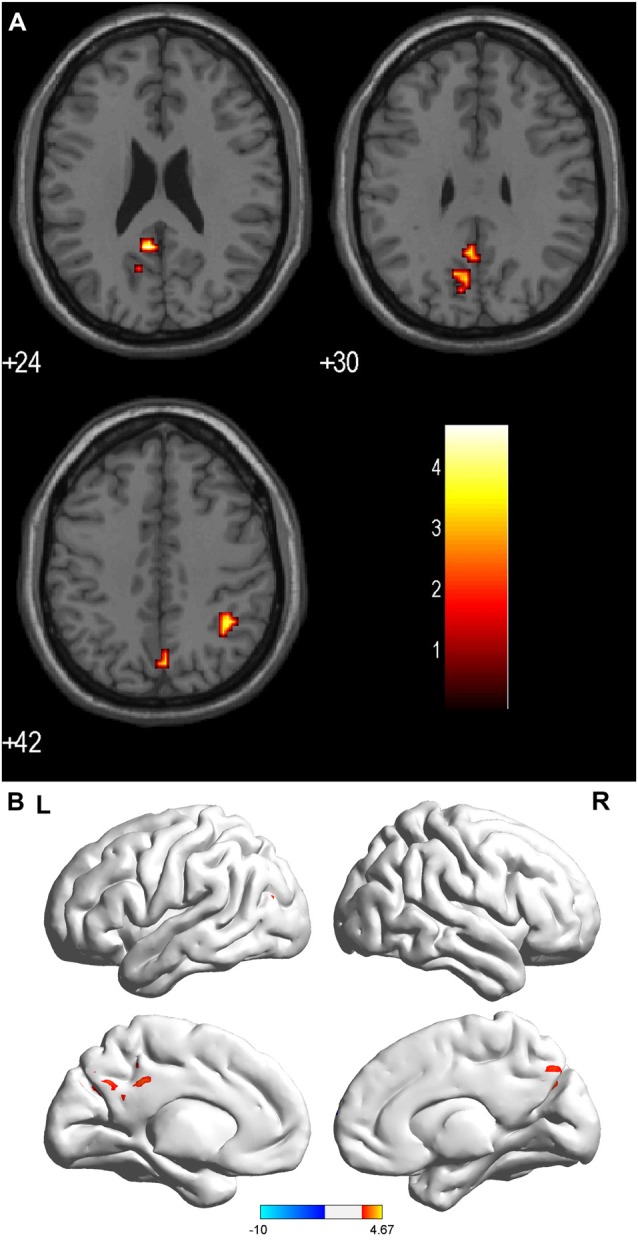
**(A,B)** ALFF: RED = sON > rON. Brain areas showing significant ALFF differences between the sON and rON patients. Notes: the different brain regions include the right IPL, left posterior cingulate, and bilateral precuneus. The red areas represent lower ALFF brain regions (corrected *p* < 0.01 using AlphaSim). Abbreviations: ON, optic neuritis; ALFF, amplitude of low-frequency fluctuation; L, left; R, right.

Compared with the HCs, the sON patients showed significantly decreased ALFFs in the bilateral cuneus/precuneus, bilateral fusiform gyrus, bilateral inferior occipital gyrus, right posterior cingulate, right parahippocampal gyrus/hippocampus, right postcentral gyrus and left superior temporal gyrus (corrected *p* < 0.01 using AlphaSim). The sON patients showed a significant increase in ALFFs in the left middle frontal gyrus, right superior frontal gyrus, right middle cingulate gyrus, left medial frontal gyrus, left precentral gyrus and left caudate (corrected *p* < 0.01 using AlphaSim; Table 2, Figures 1A,B). The rON patients demonstrated significantly decreased ALFFs in the left cuneus/precuneus, bilateral inferior temporal gyrus, bilateral lingual gyrus, right middle occipital gyrus, and left superior temporal gyrus (corrected *p* < 0.01 using AlphaSim) and increased ALFFs in the bilateral inferior gyrus and left medial frontal gyrus compared to normal controls (corrected *p* < 0.01 using AlphaSim; Table 2, Figures 2A,B). A significant decrease in ALFFs was observed in the right inferior parietal lobule (IPL), left posterior cingulate, and bilateral precuneus in the rON patients compared with the sON patients (Table 2, Figures 3A,B).

**Table 2:** Brain areas showing significant amplitude of low-frequency fluctuation (ALFF) differences among the single optic neuritis (sON), relapsing optic neuritis (rON) and normal control groups (corrected *p* < 0.01 using AlphaSim).

Anatomic area (gray matter)	MNI Coordinates			Cluster size	*T*-score
	*x*	*y*	*z*		
NC-sON					
Left Middle Frontal Gyrus	−42	30	33	1202	−5.469
Right Superior Frontal Gyrus	21	24	57	858	−5.377
Right Middle Cingulate Gyrus	0	−33	39	70	−4.492
Left Medial Frontal Gyrus	−6	54	12	45	−4.073
Left Precentral Gyrus	−45	−12	33	38	−4.192
Left Caudate	−9	3	9	26	−3.846
Bilateral Cuneus/Precuneus	0	−90	33	240	6.217
	−3	−66	60		3.584
Left Fusiform Gyrus	−45	−51	−27	150	4.807
Right Fusiform Gyrus	33	−36	−18	37	3.559
Right Inferior Occipital Gyrus	48	−81	−15	126	4.900
Left Inferior Occipital Gyrus	−24	−90	−3	103	4.457
Right Posterior Cingulate	3	−60	12	65	4.669
Right Parahippocampal Gyrus/Hippocampus	21	−9	−15	28	3.312
Right Postcentral Gyrus	39	−42	63	25	4.665
Left Superior Temporal Gyrus	−45	−33	9	22	3.994
NC-rON					
Right Inferior Frontal Gyrus	−45	39	0	1146	−4.579
Left Inferior Frontal Gyrus	−51	24	12	45	−3.965
Left Medial Frontal Gyrus	−6	45	−18	183	−4.234
	−6	54	18	73	−4.960
Left Cuneus/Precuneus	−12	−96	15	862	5.965
Right Inferior Temporal Gyrus	48	−69	−3	106	4.611
Left Inferior Temporal Gyrus	−57	−66	−6	53	5.058
Left Lingual Gyrus	−15	−60	−12	105	4.577
Right Lingual Gyrus	9	−84	0	27	3.879
Right Middle Occipital Gyrus	27	−93	0	56	4.032
Left Superior Temporal Gyrus	−57	−60	12	47	4.338
	−57	−27	9	34	4.174
Right Inferior Temporal Gyrus	63	−39	−27	21	4.251
sON-rON					
Right Inferior Parietal Lobule	39	−48	42	23	4.017
Left Posterior Cingulate	−6	−51	24	20	4.674
Left Precuneus	−6	−69	30	20	3.405
Right Precuneus	3	−72	42	17	3.296

*T-scores > 0 represent lower ALFF brain regions; T-scores < 0 represent higher ALFF brain regions*.

### Correlations Between ALFF and Clinical Data

We examined the relationships between the EDSS scores, disease duration, LogMAR scores and ALFFs. Correlations between the disease duration and ALFFs were identified in the left middle temporal gyrus, left inferior occipital gyrus, right lingual gyrus and right IPL. Significant correlations were not observed between the ALFF values and LogMAR or EDSS scores (Table 3).

**Table 3:** Correlation between disease duration and ALFF (corrected *p* < 0.05).

	Anatomic area (gray matter)	TAL Coordinates			Cluster size	*T*-score
		*x*	*y*	*z*		
Disease duration & ALFF	Left Middle Temporal Gyrus	−48	−9	−21	20	0.403
	Left Inferior Occipital Gyrus	−39	−81	−9	17	0.478
	Right Lingual Gyrus	6	−93	0	23	0.472
	Right Inferior Parietal Lobule	69	−42	21	17	0.459

## Discussion

Baseline brain activity alterations are reported in many neurological diseases, including CIS, MS and NMO (Liu et al., 2011a,b, 2012). The current study focused on evaluating the brain activity change patterns in sON and rON patients via rs-fMRI.

In the present study, we found a common pattern of decreased ALFFs in several regions of the occipital and temporal lobes in both the sON and rON patients. Wang et al. (2008) used fMRI to investigate whether spontaneous activity occurred in the primary visual areas (PVAs) of normal-sighted subjects and found that the PVA-related spontaneous activity consisted of the visual association areas including the middle occipital gyrus, cuneus and lingual gyrus, the precuneus, the precentral/postcentral gyrus, the middle frontal gyrus, the fusiform gyrus, the inferior/middle temporal gyrus and the parahippocampal gyrus. In our study, the areas with decreased ALFFs in the ON patients mainly involved the PVAs, which may be associated with memory-related mental imagery and/or visual memory consolidation processes. These changes were likely caused by axonal degeneration secondary to lesions in the optic nerves, which led to an abnormal visual pathway and impaired optic radiations related to the PVA. Both the sON and rON patients had lower ALFF values in the precuneus, which represents a key hub in the default mode network (DMN). Strong interactions have been observed between the PCC/precuneus and the IPL and medial prefrontal cortex (Buckner et al., 2008; Fransson and Marrelec, 2008). Huang et al. (2015) also found that patients with ON had lower ALFF values in the bilateral precuneus. Liu et al. demonstrated decreased ALFF values in the precuneus in CIS (Liu et al., 2012) and NMO (Liu et al., 2011a).

Compared with the HCs, significantly increased ALFFs were observed in the sON patients in the left middle frontal gyrus, right superior frontal gyrus, right middle cingulate gyrus, left medial frontal gyrus, left precentral gyrus and left caudate. The rON patients showed increased ALFFs in the bilateral inferior gyrus and left medial frontal gyrus. Previous publications revealed that visual cortical areas that selectively process relevant information are functionally connected with the frontal-parietal network, whereas those that process irrelevant information are simultaneously coupled with the default network (Chadick and Gazzaley, 2011). Our findings implied that the functional adaptations in ON occurred mainly in areas within the frontal-parietal network.

The ALFFs were also increased in the left caudate in single attack patients, and they might be involved in the control of voluntary movement, learning and memory, particularly regarding feedback processing (Packard and Knowlton, 2002). Activation of the left caudate may play important roles in adaptive neural responses after damage to both the motor and visual systems in the early stages of ON. Moreover, the inferior frontal gyrus presents increased activity in rON because it has an inverse association with the DMN (Greicius et al., 2003). The medial frontal gyrus is part of the DMN, which has a higher ALFF value in both sON and rON patients, which may reflect compensation by the DMN when patients experience visual impairment.

Interestingly, the activity of the right IPL, left posterior cingulate and bilateral precuneus were significantly lower in the rON patients compared with the sON patients, whereas regions with increased ALFFs were not identified, which demonstrated that recurrent attacks cause more severe dysfunction of the PVA. A longitudinal study found that progressive damage occurs to the optic radiations in ON (Tur et al., 2016), and this finding is consistent with the results our study, which implied both structural and functional damage progression during ON relapse.

We found positive correlations between the ALFF values and disease duration in several regions, such as the left middle temporal gyrus and inferior occipital gyrus, and these results suggest that dynamic functional plasticity changes might occur in the temporal and occipital lobes during disease progression. No other significant correlations were identified between the ALFF values and the LogMAR or EDSS scores.

### Limitations

Certain limitations were observed in this study. First, the AQP4 antibody was not observed and the ON patients were not subject to follow-up evaluations. Second, longitudinal studies must be performed to explore the brain functional alterations in the different clinical outcomes of ON patients and dynamic analysis should be done to reflect the functional plasticity. Third, the relationship between brain functional and structural changes remains unclear.

## Conclusion

In summary, functional impairments and adaptions occurred in both the sON and rON patients; however, the impairments mainly involved visual areas and functional adaptions predominantly occurred in the frontal lobe. More severe functional impairments were observed in the rON patients without any adaption compared with sON. Furthermore, the alterations in rON were correlated with the disease duration.

## Author Contributions

YL, KL and ZR: guarantor of integrity of the entire study. YL and KL: study concepts. ZR: literature research. BM and XZ: clinical studies. ZR, YD, HJ and ZS: performed the experiments. PL: data analysis and statistical analysis. ZR: manuscript preparation. ZR and YL: manuscript editing.

## Conflict of Interest Statement

The authors declare that the research was conducted in the absence of any commercial or financial relationships that could be construed as a potential conflict of interest.

## Footnotes

^1^http://www.fil.ion.ucl.ac.uk/spm

^2^http://resting-fmri.sourceforge.net

^3^http://afni.nimh.nih.gov/pub/dist/doc/manual/AlphaSim.pdf
